# CO Cross-Interference Characteristics of a Pd–Cu Fiber-Optic MEMS Hydrogen Sensor for Early Warning of Thermal Runaway in Energy Storage Batteries

**DOI:** 10.3390/s26103044

**Published:** 2026-05-12

**Authors:** Jiwei Du, Mengda Li, Yajun Jia, Junjie Jiang, Tao Liang

**Affiliations:** 1 School of Electrical Engineering, Shanghai Dianji University, Shanghai 201306, China; 18052102418@163.com; 2 School of Electrical Engineering, Shanghai Jiao Tong University, Shanghai 200240, China; 3 Faculty of Electricity, Inner Mongolia University of Technology, Hohhot 010062, China

**Keywords:** hydrogen sensor, fiber-optic MEMS hydrogen sensor, Fabry–Pérot interferometer, Pd–Cu alloy, CO cross-interference, thermal runaway early warning

## Abstract

In early-warning scenarios for thermal runaway in energy storage batteries, carbon monoxide (CO) may interfere with hydrogen detection and reduce the reliability of signal interpretation. To mitigate CO cross-interference under representative mixed-gas conditions and improve sensing stability, a fiber-optic microelectromechanical systems (MEMS) hydrogen sensor based on a Pd–Cu alloy-sensitive layer was developed. The sensor employs a single-cantilever structure and a reflective Fabry–Pérot (F–P) interferometer for optical readout. Comparative experiments were carried out using sensors coated with pure Pd and Pd–Cu-sensitive layers under pure H_2_, CO background interference, and temperature-fluctuation conditions. The Pd–Cu sensor exhibited a good linear response over 0–500 ppm H_2_, with a sensitivity of 0.0845 nm/ppm. Under a mixed atmosphere of 200 ppm H_2_ and 500 ppm CO, the Pd–Cu sensor measured 198 ppm, whereas the pure Pd sensor measured 176 ppm, corresponding to relative errors of approximately 1% and 12%, respectively. In addition, the Pd–Cu sensor showed faster response/recovery behavior and better output stability after temperature compensation. These results indicate that, under the investigated conditions, the selected Pd–Cu-sensitive layer can effectively reduce CO-induced interference and improve the accuracy and stability of fiber-optic MEMS hydrogen sensing, supporting its feasibility for representative early-warning-related monitoring scenarios in energy storage batteries.

## 1. Introduction

Hydrogen is characterized by rapid diffusion, low ignition energy, and a wide explosive range. Once released into a practical environment and accumulated to a critical concentration, it can readily cause combustion or even explosion [1]. In recent years, with the widespread application of energy storage batteries in renewable energy integration, electric transportation, and large-scale energy storage systems, their safety issues, especially the risk of thermal runaway, have attracted increasing attention [2]. Thermal runaway is typically accompanied by intense heat generation, characteristic gas release, and the chain amplification of side reactions, which may ultimately lead to fire or explosion. Therefore, rapid, accurate, and reliable monitoring of early-warning signals is of great significance for improving the safety warning capability of energy storage systems [3].

During the early evolution of thermal runaway in energy storage batteries, H_2_, CO, CO_2_, and small amounts of hydrocarbons are gradually released [4]. Among these gases, hydrogen is usually released at an early stage and can be detected at concentrations ranging from tens to hundreds of ppm; it is therefore regarded as one of the most promising characteristic gases for early warning [5,6]. However, hydrogen monitoring under practical operating conditions is not merely a single-gas identification problem. CO is not only a common gaseous product generated during battery thermal runaway, but may also coexist with H_2_ in actual battery venting, thereby causing cross-interference in hydrogen detection and increasing the difficulty of signal interpretation [7]. Therefore, hydrogen sensing for the early warning of battery thermal runaway requires not only high sensitivity, but also good accuracy and anti-interference capability under complex background atmospheres [8]. In addition to H_2_ and CO, battery thermal runaway may also release CH_4_, VOC_s_, and other gaseous species. In the present work, CO was selected as a representative interference gas to evaluate the anti-interference capability of the sensor under a simplified yet practically relevant mixed-gas condition.

At present, hydrogen detection technologies mainly include electrochemical, semiconductor, catalytic combustion, and optical methods. Among them, optical methods, especially fiber-optic hydrogen sensors, have attracted considerable attention because of their immunity to electromagnetic interference, intrinsic safety, suitability for remote interrogation, and potential for miniaturization and integration [9,10]. Among various fiber-optic hydrogen-sensing configurations, Fabry–Pérot (F–P) interferometric sensors are particularly attractive because changes in the cavity optical path difference can be converted into measurable spectral variations [11]. In hydrogen-sensitive F–P devices, these variations are often associated with hydrogen-induced deformation of a compliant microstructure, which changes the cavity length and shifts the interference spectrum [12,13]. These reports indicate that combining hydrogen-sensitive materials with microstructured mechanical amplification is a promising strategy for improving fiber-optic hydrogen sensing performance [14].

In such fiber-optic hydrogen sensing systems, Pd and its alloys are widely used as sensitive materials because of their excellent catalytic dissociation activity and hydrogen absorption capability [15]. However, pure Pd still suffers from several limitations. It is prone to hysteresis and structural instability during hydrogen absorption and desorption, which degrades the repeatability and long-term reliability of the sensor [16,17]. In addition, the preferential adsorption of CO on the Pd surface occupies the active sites required for hydrogen dissociation and adsorption and suppresses hydrogen absorption and diffusion within the sensitive layer, thereby leading to weakened response, slower recovery, and even output drift [18]. Therefore, systematically investigating CO cross-interference and regulating the surface and bulk behavior of Pd-sensitive layers through alloying are considered important approaches for improving the environmental adaptability of Pd-based hydrogen sensors under representative interference conditions [19].

Although fiber-optic hydrogen sensing has developed rapidly in recent years, most reported studies still focus mainly on basic performance evaluation under pure H_2_ conditions, such as response amplitude, sensitivity, and response/recovery time [20]. However, for fiber-optic MEMS hydrogen sensors driven by thin-film strain, performance assessment based solely on pure hydrogen response is insufficient for practical use because sensing stability under background-gas interference and temperature fluctuation also plays an important role in early-warning-related monitoring performance. In representative application scenarios such as the early warning of thermal runaway in energy storage batteries, the sensor should also exhibit strong anti-interference capability under CO and other background gases, good stability under temperature fluctuations, and high reliability during long-term operation [21]. In particular, when CO coexists with H_2_, the choice of sensitive material directly affects the detection accuracy and environmental adaptability of the sensor. Therefore, improving the CO tolerance of Pd-based sensitive layers through material modification has become an important issue in the development of highly reliable hydrogen sensors.

Based on this motivation, a fiber-optic MEMS hydrogen sensor incorporating a Pd–Cu-sensitive layer was developed in this work, and the effect of alloying on sensing performance was investigated with a particular focus on CO cross-interference. Two types of sensors coated with pure Pd and Pd–Cu-sensitive layers were fabricated and comparatively evaluated under pure H_2_, CO background interference, and temperature-fluctuation conditions. The differences in dynamic response, sensing deviation, hysteresis behavior, and stability between the two sensitive materials were analyzed. The results provide experimental evidence that the selected Pd–Cu-sensitive layer can improve hydrogen sensing performance relative to pure Pd under the investigated conditions and support its potential use in representative early-warning-related sensing scenarios for energy storage batteries.

## 2. Materials and Methods

When the Pd-based sensitive layer is exposed to a hydrogen-containing atmosphere, hydrogen molecules are first dissociatively adsorbed on the Pd surface and then diffuse into the lattice, generating lattice expansion and internal stress in the sensitive layer [22]. In cantilever-based structures, this hydrogen-induced stress leads to bending deformation of the microcantilever [23]. In the present Fabry–Pérot device, the resulting mechanical displacement changes the cavity length and is further converted into a shift in the interference spectrum, thereby enabling optical readout of the hydrogen signal [24].

**Figure 1:**
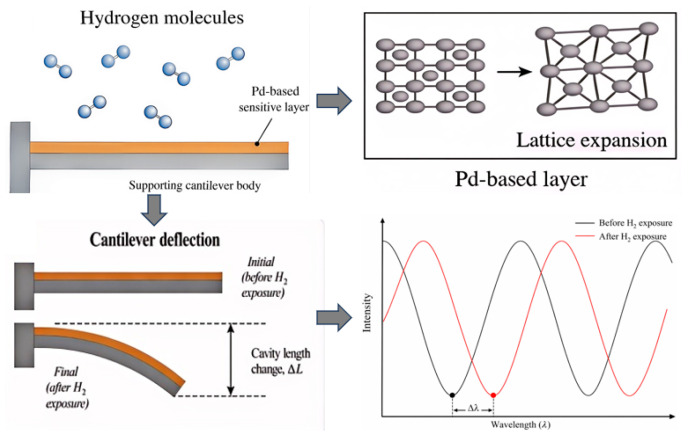
Schematic diagram illustrating the mechanism of hydrogen adsorption-induced cantilever deflection and spectral drift.

Figure 1 illustrates the sensing mechanism of the proposed sensor. Hydrogen-induced expansion of the sensitive layer generates bending deformation of the cantilever, which changes the Fabry–Pérot cavity length and consequently shifts the interference spectrum.

**Figure 2:**
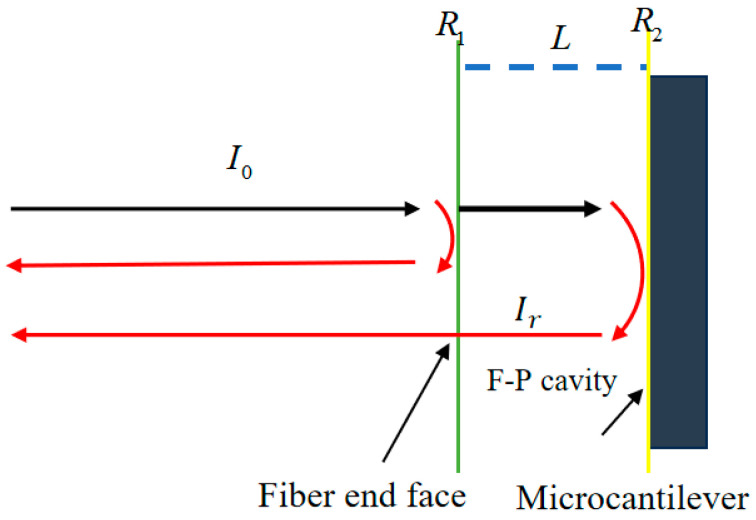
Schematic diagram of the structure and operating principle of a reflective Fabry-Pérot interferometer.

As shown in Figure 2, the reflective Fabry–Pérot (F–P) cavity is formed by two parallel reflecting surfaces, namely the fiber end face and the reflective surface of the microcantilever. The reflectance of the fiber end face is denoted by *R*_1_, the reflectance of the cantilever surface is denoted by *R*_2_, and the cavity length between the two reflecting surfaces is *L*. When the incident light *I*_0_ is transmitted through the optical fiber to the sensor head, part of it is reflected at the fiber end face, while the remaining light enters the cavity and is reflected again by the microcantilever surface before returning to the fiber. The phase difference between the two reflected beams can be expressed as [25]:

(1) $$\Delta \varphi =\frac{4\pi nL}{\lambda }\;$$

where n is the refractive index of the cavity medium, L is the cavity length, and λ is the wavelength of the incident light.

For the present reflective cavity, a simplified two-beam interference model is adopted to describe the spectral response in a qualitative manner. When the effective reflectivities of the two interfaces are relatively low, the contribution of higher-order multiple reflections inside the cavity can be neglected, and the reflected intensity can be approximated as follows [26]:

(2) $$I_{r}=I_{0}(R_{1}+R_{2}-2\sqrt{R_{1}R_{2}}cos\frac{4\pi nL}{\lambda })$$

The applicability of the simplified two-beam approximation was further evaluated according to the optical characteristics of the reflective F–P cavity used in this work. In the proposed sensor, the first reflecting interface is the cleaved silica fiber end face in air, whose reflectivity can be estimated using the Fresnel equation:

(3) $$R_{1}={\left(\frac{n_{f}-n_{air}}{n_{f}+n_{air}}\right)}^{2}$$

where *n_f_* and *n_air_* are the refractive indices of the silica fiber and air, respectively. Taking *n_f_* ≈ 1.444 and *n_air_* ≈ 1.000 near 1550 nm, the reflectivity of the fiber end face is approximately 0.033–0.035. The second reflecting interface is the Au-coated cantilever surface. Considering the finite thickness of the Au reflective layer, beam divergence, surface roughness, and possible alignment loss in the packaged sensor, the effective reflectivity of this interface was estimated to be approximately 0.5 for the present order-of-magnitude analysis.

For a reflective Fabry–Pérot cavity, the reflected intensity can be generally described by an Airy-type expression:

(4) $$I_{r}=I_{0}\frac{R_{1}+R_{2}-2\sqrt{R_{1}R_{2}}\cos\left(\frac{4\pi nL}{\lambda }\right)}{1+R_{1}R_{2}-2\sqrt{R_{1}R_{2}}\cos\left(\frac{4\pi nL}{\lambda }\right)}$$

where *R*_1_ and *R*_2_ are the reflectivities of the two reflecting interfaces, *L* is the cavity length, *n* is the refractive index of the cavity medium, and *λ* is the incident wavelength. In the present device, *R*_1_ is only approximately 3.5%, and the product *R*_1_*R*_2_ is approximately 0.0175 when *R*_2_ ≈ 0.5. Therefore, the contribution of higher-order multiple reflections is relatively limited, and the cavity can be regarded as a low-finesse reflective F–P cavity. Under this condition, the simplified two-beam model provides an appropriate approximation for describing the phase-dependent spectral modulation and tracking the wavelength shift in the characteristic interference dip.

It should be noted that Equation (2) is not used to reconstruct the complete intracavity optical field or to fit the absolute reflected intensity. Instead, it is used to describe the relationship between cavity-length variation and spectral wavelength shift. Since the sensing response in this work is obtained by tracking the wavelength shift of a selected interference dip, the wavelength variation is mainly governed by the phase term 4*πnL*/*λ*. When hydrogen absorption in the Pd–Cu-sensitive layer induces cantilever deflection, the F–P cavity length changes accordingly, resulting in a measurable shift in the reflection spectrum. Therefore, the simplified model is appropriate for wavelength-demodulated sensing analysis under the low-finesse cavity condition adopted in this work.

**Figure 3:**
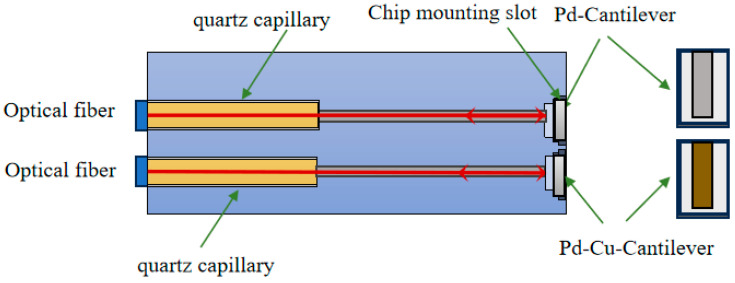
Schematic diagram of the fiber-optic MEMS hydrogen-sensitive sensor structure.

To compare the effects of different sensitive materials on sensor performance under similar optical and packaging conditions, a dual-channel structure was designed, as shown in Figure 3.

The sensor mainly consists of optical fibers, quartz capillaries, chip mounting slots, and sensitive chips. The two optical fiber channels adopt the same structural configuration and are connected to microcantilever chips coated with pure Pd and Pd–Cu-sensitive layers, respectively, allowing direct comparison of sensor performance under consistent optical and packaging conditions.

**Figure 4:**
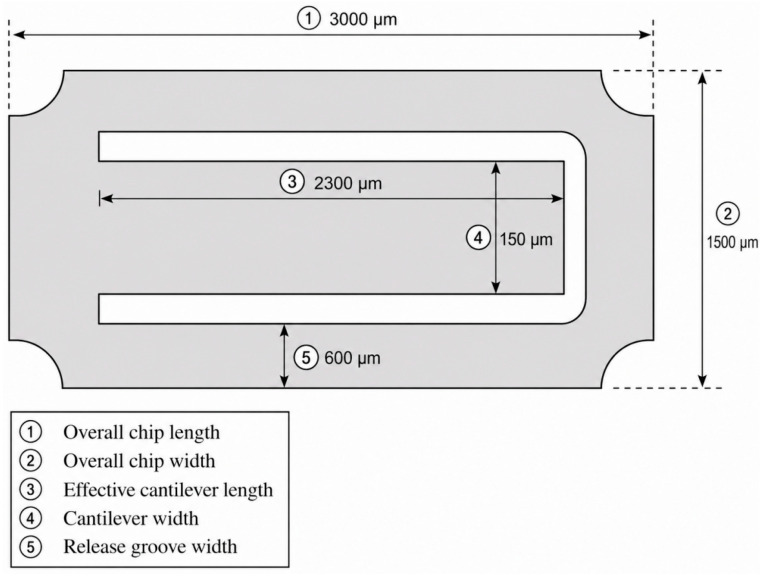
Planar geometry and key dimensions of the microcantilever chip.

The planar geometry and key dimensions of the microcantilever chip are shown in Figure 4.

Figure 4 shows the planar geometry and key dimensions of the microcantilever chip used in the proposed fiber-optic MEMS hydrogen sensor. The overall chip size is approximately 3000 μm × 1500 μm. The effective length of the main cantilever region is about 2300 μm, the cantilever width is 150 μm, and the width of the etched release groove is approximately 600 μm. The release groove provides sufficient space for cantilever deformation under the strain generated by the sensitive layer, while helping to maintain the structural integrity of the device.

The sensing unit adopts a Pd–Cu/Si/Au multilayer structure, in which a 60 nm Pd–Cu alloy film with a designed Pd-to-Cu ratio of 7:3 serves as the hydrogen-sensitive layer, a 10 μm Si layer acts as the supporting layer, and a 70 nm Au layer functions as the reflective layer. The introduction of Cu is intended to reduce the CO cross-interference commonly observed in pure Pd films while preserving hydrogen-induced strain generation. In this way, the multilayer design provides the structural basis for reflective fiber-optic F–P hydrogen sensing with improved resistance to CO interference.

The Pd-to-Cu ratio of 7:3 was selected as a designed alloy composition for preliminary evaluation rather than as an optimized composition. This ratio was used to investigate whether Cu alloying could reduce CO-related interference while retaining the hydrogen-induced strain response of the Pd-based sensitive layer. In the present work, both the pure Pd and Pd–Cu sensors were fabricated using the same cantilever geometry, film-thickness design, and packaging process, so that the influence of the sensitive-layer composition could be compared under consistent structural and optical conditions. The designed Pd:Cu composition was controlled during magnetron co-sputtering by adjusting the relative sputtering powers of the Pd and Cu targets, and the film thickness was monitored using a quartz crystal microbalance. Therefore, the reported Pd–Cu response should be interpreted as the performance of a selected representative Pd–Cu alloy layer under the investigated conditions, rather than as the result of systematic composition optimization.

The chip was fabricated using a silicon-based MEMS process. First, the predefined cantilever pattern was transferred onto the silicon substrate by photolithography to form the etching mask. The cantilever profile was then defined by front-side deep reactive ion etching (DRIE), while the supporting region beneath the cantilever was removed by back-side DRIE followed by structural release, resulting in a single-cantilever chip with a free end. This fabrication process produced a well-defined cantilever structure with clear boundaries and good uniformity, providing a reliable platform for subsequent deposition of the Pd–Cu-sensitive layer and optical fiber packaging. Since the main objective of this study was to evaluate the influence of sensitive-layer composition on hydrogen sensing performance under CO background conditions, the same chip structure and packaging strategy were used for both pure Pd and Pd–Cu sensors, so that the effect of the sensitive-layer composition on CO cross-interference could be directly compared under consistent structural and optical conditions.

**Figure 5:**
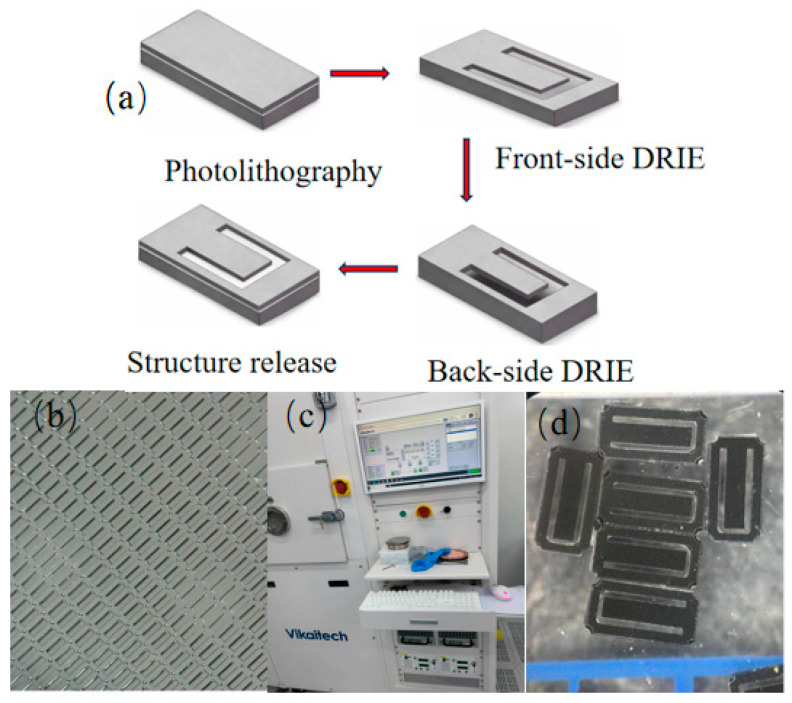
Chip fabrication process and physical images: (**a**) shows a schematic diagram of the chip fabrication process; (**b**) shows a microscopic image of the cantilever array; (**c**) shows the DRIE processing equipment; and (**d**) shows a physical image of the chip.

Figure 5 presents the main fabrication steps and representative photographs of the chip. Figure 5a shows the schematic fabrication process, Figure 5b shows the microscopic image of the cantilever array, Figure 5c shows the DRIE system used for chip processing, and Figure 5d shows the fabricated chip.

To suppress the CO cross-interference commonly observed in pure Pd films, a Pd–Cu alloy layer was deposited on the cantilever surface by magnetron co-sputtering. Before deposition, the chamber was evacuated to below 1.0 × 10^−4^ Pa, followed by a 5–10 min pre-sputtering step to remove surface contamination from the targets. Ar was used as the working gas during deposition. The designed Pd:Cu ratio was controlled by adjusting the relative sputtering powers of the Pd and Cu targets. During deposition, the film thickness was monitored in real time using a quartz crystal microbalance (QCM), and the deposition was terminated when the target thickness was reached. In this way, a Pd–Cu-sensitive layer with a thickness of about 60 nm was formed on the cantilever surface. Subsequently, a Au film with a thickness of approximately 70 nm was deposited on the reflective surface, and its thickness was controlled in the same manner, so as to enhance the optical reflectivity and improve the quality of the interference signal.

**Figure 6:**
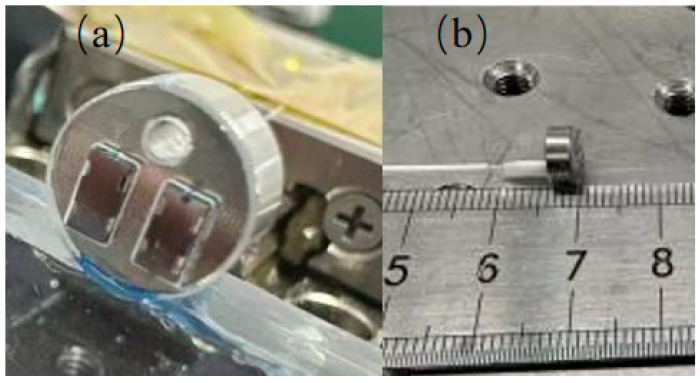
Photographs of the fiber-optic MEMS hydrogen-sensitive sensor package: (**a**) shows a close-up view of the chip after mounting, whilst (**b**) shows the overall appearance of the sensor after packaging.

After chip fabrication and sensitive-layer deposition, the sensor was packaged as shown in Figure 6.

During packaging, a reflective Fabry–Pérot (F–P) cavity was formed by adjusting the relative position between the fiber end face and the reflective surface of the cantilever. The fiber was aligned approximately normal to the cantilever reflector, and the cavity length was tuned until a clear interference spectrum with good fringe visibility was obtained. After alignment, the optical fiber held in the quartz capillary and the mounted chip were fixed by the packaging structure to maintain a stable Fabry–Pérot cavity during measurement.

**Figure 7:**
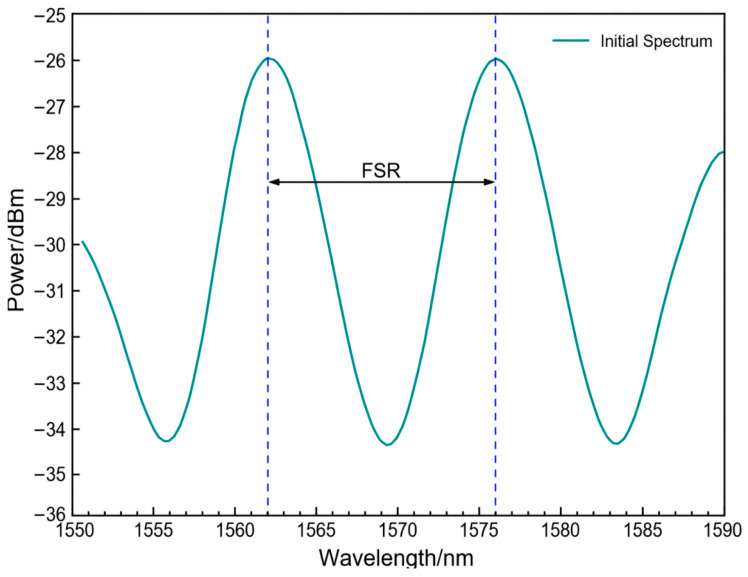
Initial reflection spectrum of the packaged sensor used for estimation of the Fabry–Pérot cavity length. The free spectral range (FSR) was extracted from two adjacent interference maxima.

The initial reflection spectrum of the packaged sensor is shown in Figure 7, from which the free spectral range (FSR) was determined from the wavelength interval between two adjacent interference maxima. For the air-filled F–P cavity, the cavity length *L* can be estimated from the FSR as follows:

(5) $$L=\frac{\lambda^{2}}{2n\cdot \mathrm{FSR}}$$

where n is the refractive index of the cavity medium and λ is the central wavelength. Based on the measured FSR, the initial cavity length of the packaged sensor was estimated to be approximately 85 μm. This cavity length provided good interference visibility and appropriate fringe spacing within the measurement wavelength range, thereby facilitating wavelength demodulation based on characteristic spectral shift.

**Figure 8:**
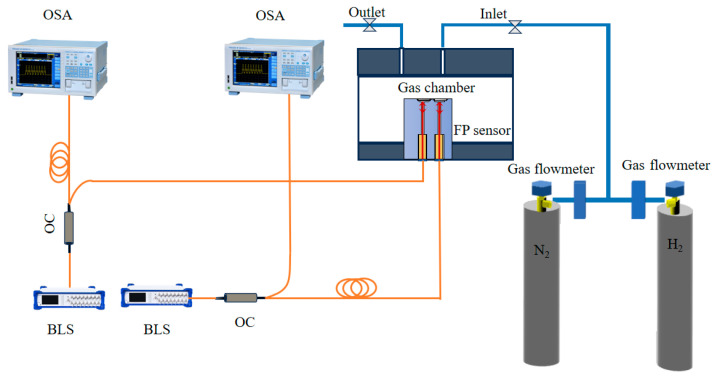
Block diagram of the fiber-optic MEMS hydrogen-sensitive sensing system.

Figure 8 shows the schematic of the fiber-optic MEMS hydrogen sensing system. The experimental platform mainly consists of two identical optical interrogation channels, each including a broadband light source, an optical circulator, and a spectrometer, together with a sealed gas chamber and a gas delivery unit. The reflection spectra of the two sensors were acquired in real time by the spectrometers, and all tests under different gas conditions were carried out on this platform. Since each spectrometer could interrogate only one sensing channel, two identical spectrometers were employed in the dual-channel system. The pure Pd sensor and the Pd–Cu sensor were connected to two independent but identical optical channels and tested simultaneously in the same chamber under identical gas and temperature conditions, thereby enabling controlled comparison of the two sensitive layers.

Gas-sensing measurements were performed in a sealed chamber with an internal volume of approximately 5 L. Standard H_2_/N_2_ and CO/N_2_ gas mixtures were introduced through the gas delivery unit to obtain the required test atmospheres, and the inlet flow condition was kept consistent between different exposure and purge stages using gas flowmeters. The pure Pd and Pd–Cu sensors were placed in the same chamber and tested simultaneously, ensuring identical gas composition, temperature, exposure duration, and purge conditions. Unless otherwise specified, the tests were conducted at 25 ± 1 °C under dry-gas conditions. Before each exposure step, the chamber was purged with pure N_2_ until the characteristic interference wavelength returned to a stable baseline. Each exposure or purge stage was maintained for approximately 20 min, allowing the sensor output to approach a stable plateau.

The reflection spectra were acquired using two identical YOKOGAWA AQ6370E optical spectrum analyzers (Yokogawa Test & Measurement Corporation, Tokyo, Japan) with a spectral resolution of 0.02 nm. The characteristic wavelength was extracted as the minimum wavelength of a selected interference dip, and the same dip was tracked throughout the calibration, dynamic response, CO-interference, and temperature-compensation experiments. The main experimental conditions are summarized in Table 1.

## 3. Results

The hydrogen concentration range of 0–500 ppm was selected to cover the low-concentration regime relevant to early-stage hydrogen release, when early warning capability is of primary interest. Within this range, the sensor’s performance in terms of sensitivity, linearity, and interference resistance can be evaluated more effectively than at excessively high concentrations. A concentration of 200 ppm H_2_ was used in the dynamic and mixed-gas tests because it represents an intermediate level within the calibration range and allows the response variation under different interference conditions to be compared more clearly. The CO concentration of 500 ppm was chosen as a relatively strong interference background in order to evaluate the anti-interference capability of the sensor under a stringent test condition.

To evaluate the sensing performance of the proposed sensor under different operating conditions, the results are presented in four parts. First, the hydrogen response and calibration behavior of the Pd–Cu sensor under pure H_2_ are examined. Next, the dynamic response and repeatability of the pure Pd and Pd–Cu sensors are compared. The CO cross-interference performance is then analyzed as the main focus of this study. Finally, the temperature-dependent response and compensation results are presented to assess sensor stability under representative coupled gas–temperature conditions.

### 3.1. Hydrogen Response and Calibration

At 25 °C, step-response tests were carried out on the Pd–Cu fiber-optic MEMS hydrogen sensor using standard H_2_/N_2_ gas mixtures with hydrogen concentrations ranging from 0 to 500 ppm.

**Figure 9:**
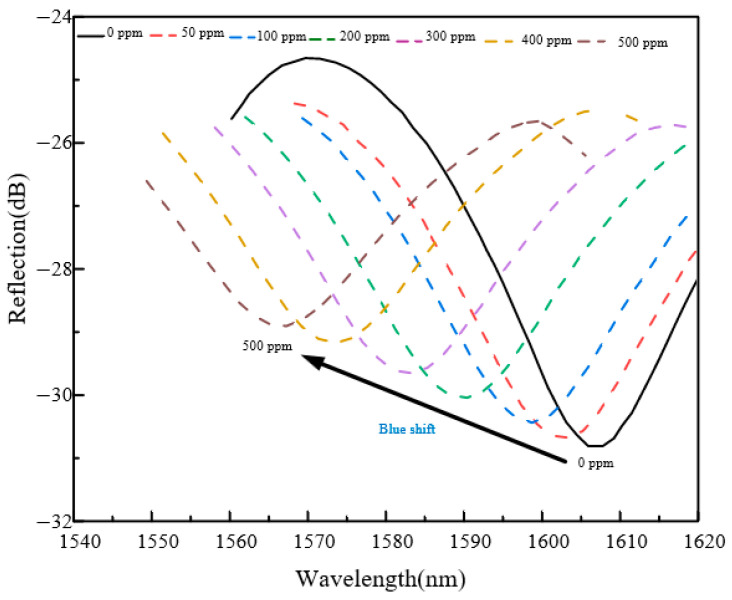
Reflection spectral shifts at different H_2_ concentrations.

**Figure 10:**
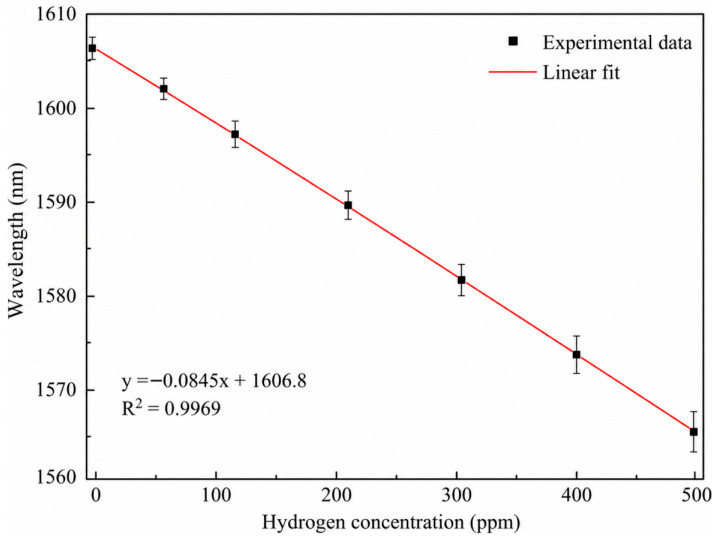
Linear calibration of the characteristic wavelength as a function of H_2_ concentration. Error bars represent the standard deviations of repeated wavelength extractions from the stable plateau region.

Figure 9 and Figure 10 summarize the spectral response and calibration behavior of the Pd–Cu sensor under different H_2_ concentrations. As shown in Figure 10, the characteristic wavelength decreases linearly with increasing H_2_ concentration over 0–500 ppm, with a fitted sensitivity of −0.0845 nm ppm^−1^ and a coefficient of determination of 0.9969. The error bars represent the standard deviations of repeated characteristic-wavelength extractions from the stable plateau region at each concentration, indicating good repeatability of the wavelength-demodulated calibration.

### 3.2. Dynamic Response and Repeatability

To compare the dynamic response characteristics of sensors with Pd–Cu and Pd sensitive layers, a three-cycle test was carried out at 25 °C using 200 ppm H_2_. During the test, the sensors were alternately exposed to 200 ppm H_2_/N_2_ and pure N_2_, with each stage lasting 20 min. Here, pure N_2_ was used as the baseline and purge gas during the recovery stage to remove residual H_2_ from the chamber and restore the sensor signal toward the baseline between consecutive exposure steps.

**Figure 11:**
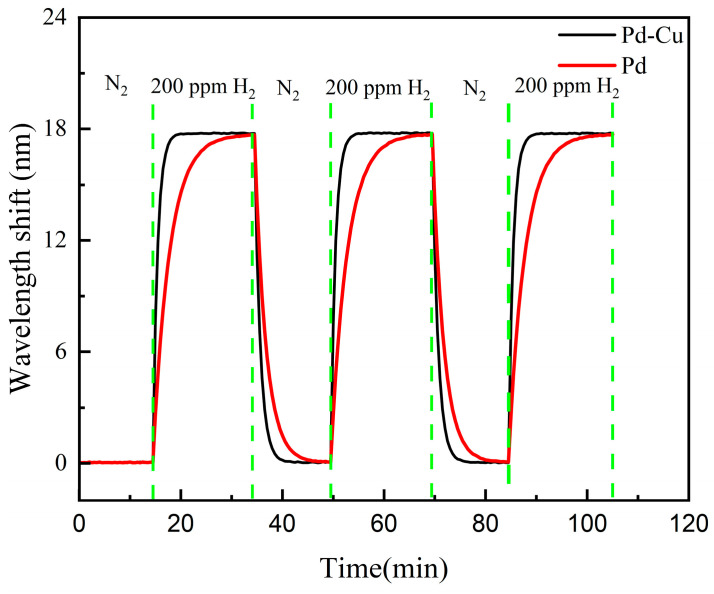
Three-cycle dynamic response and recovery curves of the Pd–Cu and pure Pd sensors under alternating 200 ppm H_2_/N_2_ and pure N_2_ atmospheres.

The three-cycle dynamic responses in Figure 11 and the statistical results in Table 2 were used to evaluate the repeatability and dynamic characteristics of the two sensors. Both sensors maintained reversible responses under alternating pure N_2_ and 200 ppm H_2_/N_2_ atmospheres. Compared with the pure Pd sensor, the Pd–Cu sensor reached the response and recovery plateaus more rapidly and showed smaller cycle-to-cycle fluctuation. Using the 90% full-scale criterion, the average response and recovery times of the Pd–Cu sensor over the three exposure–purge cycles were 3.6 ± 0.3 min and 3.1 ± 0.2 min, respectively, whereas those of the pure Pd sensor were 7.6 ± 0.4 min and 5.5 ± 0.3 min, respectively. The cycle-to-cycle RSD values were 2.1% for the Pd–Cu sensor and 3.8% for the pure Pd sensor, further indicating the better repeatability of the Pd–Cu sensor during repeated hydrogen adsorption/desorption processes.

### 3.3. CO Cross-Interference Performance

Comparative response tests were then carried out using sensors with Pd–Cu and Pd sensitive layers under different gas conditions.

**Figure 12:**
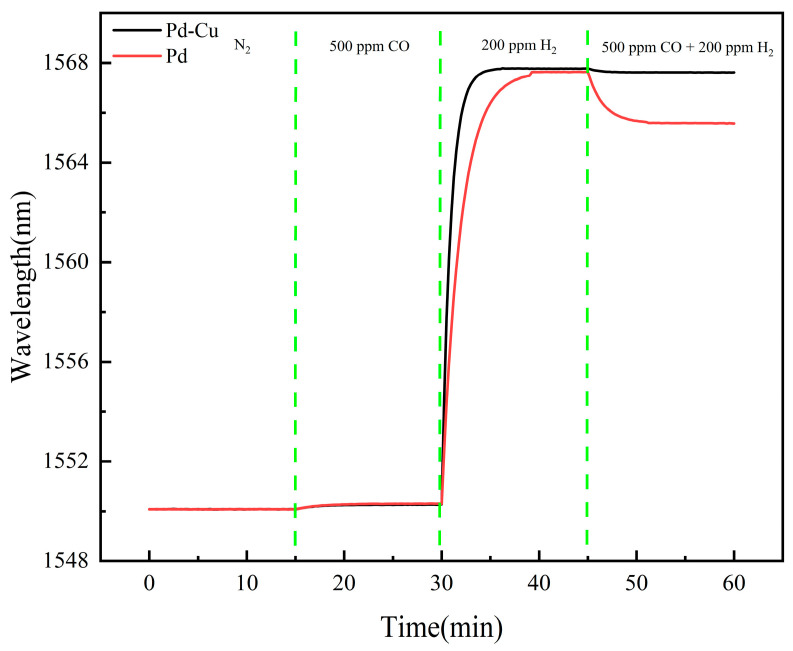
Dynamic response curves of the Pd–Cu and pure Pd sensors under sequential exposure to N_2_, 500 ppm CO, 200 ppm H_2_, and 200 ppm H_2_ + 500 ppm CO.

**Figure 13:**
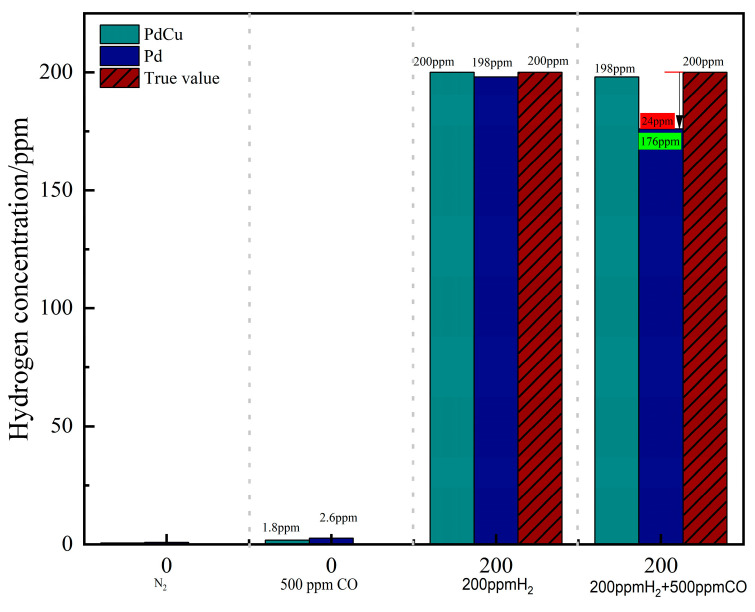
Comparison of steady-state outputs of the two sensors under different gas conditions.

Figure 12 compares the dynamic responses of the Pd and Pd–Cu sensors under different gas conditions. Both sensors remained close to the baseline under pure N_2_ and 500 ppm CO, whereas clear responses were observed under 200 ppm H_2_. Under the mixed-gas condition of 200 ppm H_2_ + 500 ppm CO, the Pd–Cu sensor maintained a response close to that obtained under pure H_2_, while the pure Pd sensor exhibited a noticeable signal decrease. This comparison indicates that the Pd–Cu-sensitive layer more effectively suppressed the influence of CO on hydrogen detection under the tested mixed-gas condition. The steady-state outputs obtained from the same experiments are quantitatively summarized in Figure 13.

As summarized in Figure 13, both sensors remained essentially stable under pure N_2_ and 500 ppm CO, while distinct responses were observed under 200 ppm H_2_. Under the mixed-gas condition of 200 ppm H_2_ + 500 ppm CO, the Pd–Cu sensor gave a measured value of approximately 198 ppm, whereas the pure Pd sensor yielded about 176 ppm. Relative to the true hydrogen concentration of 200 ppm, the Pd–Cu sensor showed a much smaller deviation, indicating improved resistance to CO interference under the present test condition.

### 3.4. Temperature-Dependent Response and Compensation

**Figure 14:**
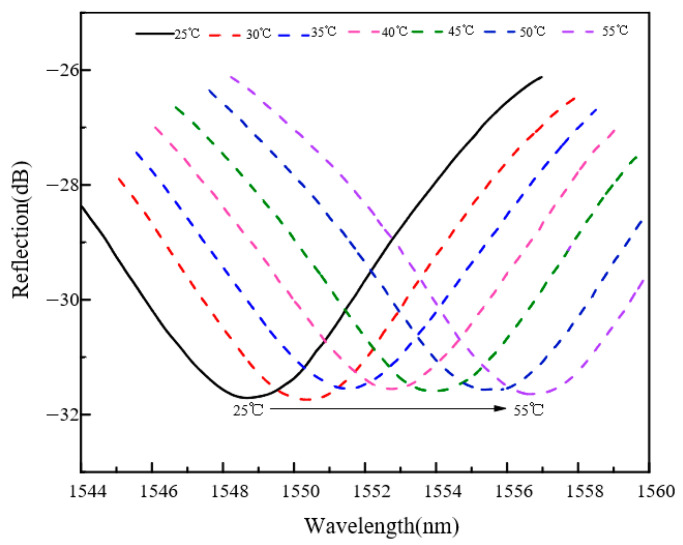
Temperature response characteristics of the sensor with a pure Pd-sensitive layer.

**Figure 15:**
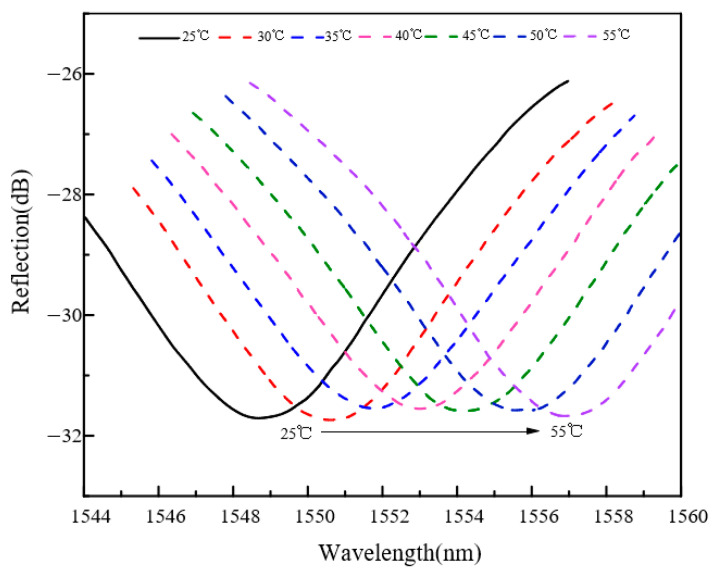
Temperature response characteristics of the sensor with a Pd–Cu-sensitive layer.

The temperature-dependent spectral shifts shown in Figure 14 and Figure 15 provide the basis for the temperature calibration and compensation procedure described below. Figure 14 and Figure 15 compare the temperature response characteristics of sensors with pure Pd and Pd–Cu-sensitive layers, respectively. Within the same temperature range, the Pd–Cu sensor exhibits a larger spectral shift than the pure Pd sensor, indicating a stronger temperature-dependent response. This behavior may be related to differences in the thermal expansion characteristics of the sensitive layers and their mechanical coupling with the cantilever structure.

To quantify the temperature effect, temperature calibration was first performed by tracking the characteristic dip wavelength in the range of 25–55 °C. Taking 25 °C as the reference, the temperature-induced wavelength shift was expressed as:

(6) $$\Delta \lambda_{T}=\lambda_{T}-\lambda_{25^{\circ }C}$$

where λ_T_ is the characteristic wavelength at temperature T. Based on the measured spectral shifts at different temperatures, the temperature-induced contribution to the sensor output was quantified for each sensing structure.

**Figure 16:**
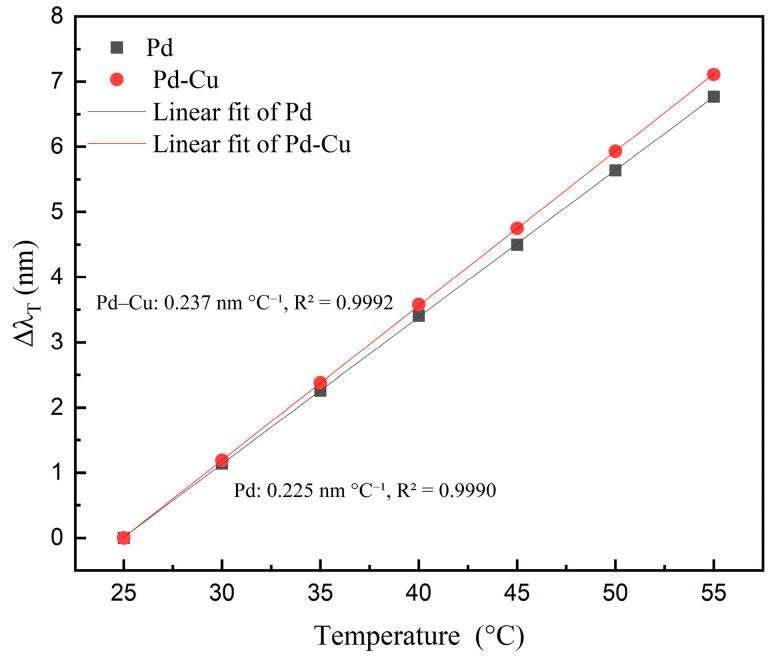
Temperature calibration curves of the pure Pd and Pd–Cu sensors based on the relative wavelength shift of the selected interference dip.

Figure 16 further summarizes the temperature calibration results extracted from the characteristic interference dips in Figure 14 and Figure 15. By using the wavelength shift relative to 25 °C, the influence of the initial cavity-length difference between the two independent sensing channels was avoided. Both sensors exhibited a monotonic red shift with increasing temperature, and the temperature-induced wavelength shift showed good linearity in the range of 25–55 °C. The fitted temperature sensitivities were 0.225 nm °C^−1^ for the pure Pd sensor and 0.237 nm °C^−1^ for the Pd–Cu sensor, with coefficients of determination of 0.9990 and 0.9992, respectively. The slightly higher temperature sensitivity of the Pd–Cu sensor may be attributed to the stronger thermal-expansion mismatch introduced by Cu alloying. However, since the measured temperature response is jointly affected by multilayer thermal expansion, cantilever deformation, cavity-length variation, and optical-interface effects, the temperature-induced wavelength drift was treated as an overall experimental correction term in the following compensation procedure.

The residual wavelength error was estimated from the difference between the measured temperature-induced wavelength shift and the corresponding fitted value. As summarized in Table 3, the maximum residual wavelength errors of both sensors were below 0.05 nm, corresponding to equivalent H_2_ concentration errors below 0.6 ppm according to the H_2_ sensitivity of 0.0845 nm ppm^−1^. This indicates that the temperature calibration provided a reasonable basis for reducing the dominant temperature-induced drift, although residual temperature-related uncertainty may still remain.

Since the hydrogen calibration curve under pure H_2_ conditions has already been established in Section 3.1, the measured wavelength shift under coupled temperature–gas conditions was corrected by subtracting the temperature-induced component, i.e.,

(7) $$\Delta \lambda_{H_{2},corr}=\Delta \lambda_{meas}-\Delta \lambda_{T}$$

The compensated wavelength shift was then converted into the equivalent hydrogen concentration using the calibration relationship obtained under pure hydrogen conditions. In the present work, the temperature effect is treated as the overall experimentally measured spectral drift. Possible secondary optical effects of the Fabry–Pérot cavity, including thermo-optic and reflectivity-related contributions, were not separated individually but were incorporated into the temperature calibration results.

To further evaluate detection stability under temperature–gas coupling conditions, heating–cooling cycle experiments were carried out in a gas mixture of 200 ppm H_2_ and 500 ppm CO.

**Figure 17:**
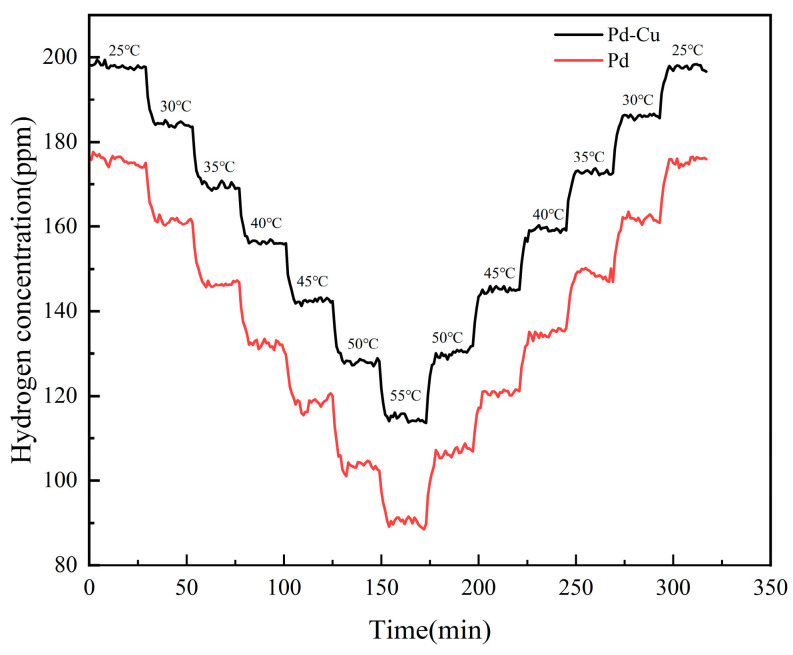
Heating–cooling responses of sensors with different sensitive layers under 200 ppm H_2_ + 500 ppm CO.

Figure 17 indicates that temperature drift cannot be neglected under the mixed-gas condition. Therefore, compensation was performed using the temperature calibration results obtained above.

**Figure 18:**
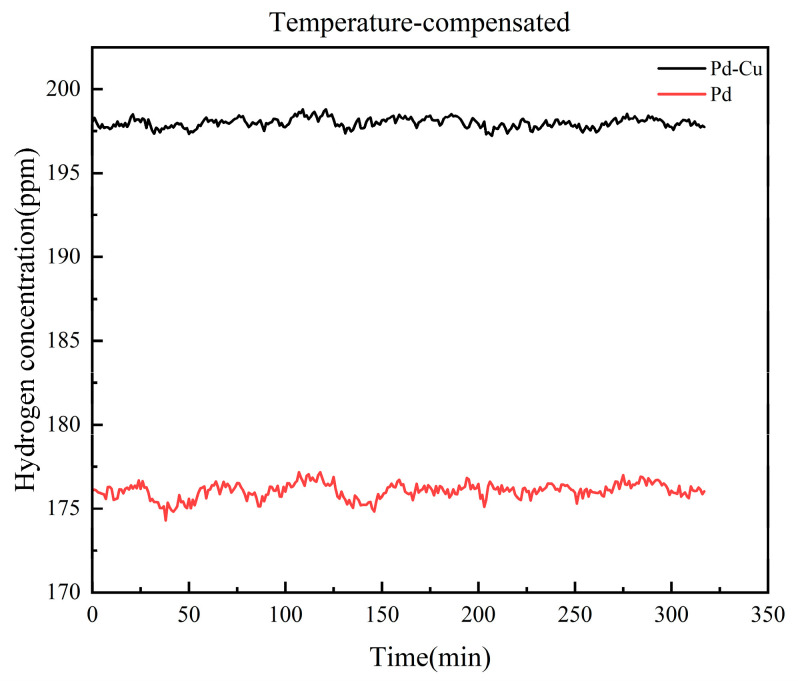
Temperature-compensated dynamic outputs of sensors with different sensitive layers under 200 ppm H_2_ + 500 ppm CO.

After compensation using the sensor-specific temperature calibration results, the dynamic outputs of both sensors became significantly more stable, as shown in Figure 18, indicating that the temperature-induced drift was effectively reduced. However, a clear difference between the two sensing materials still remained. The compensated Pd–Cu sensor maintained an output close to the target concentration, whereas the compensated pure Pd sensor still showed a noticeable underestimation.

**Figure 19:**
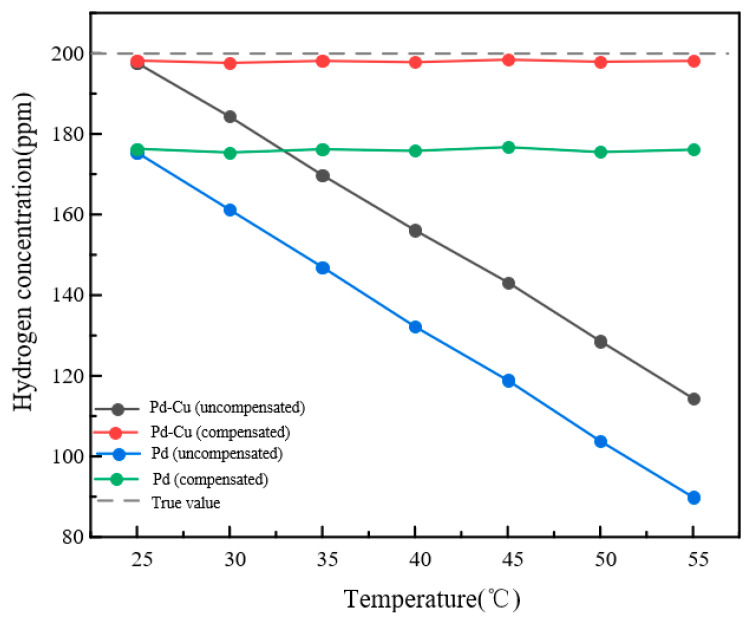
Steady-state outputs of sensors with different sensitive layers before and after temperature compensation.

Figure 19 further confirms that the compensated Pd–Cu sensor remains close to the true concentration over the tested temperature range. In contrast, although temperature compensation significantly reduces the drift of the pure Pd sensor, its output still remains lower than the true value. These results indicate that the selected Pd–Cu-sensitive layer not only suppresses CO cross-interference more effectively, but also provides better output stability under coupled temperature–gas conditions.

## 4. Conclusions

A fiber-optic MEMS hydrogen sensor based on a Pd–Cu alloy-sensitive layer was developed and experimentally evaluated under representative CO-interference conditions. The sensor showed a good linear response in the range of 0–500 ppm H_2_, with a sensitivity of 0.0845 nm ppm^−1^ and a coefficient of determination of 0.9969. Compared with the pure Pd sensor, the Pd–Cu sensor produced an output much closer to the true hydrogen concentration under the mixed atmosphere of 200 ppm H_2_ + 500 ppm CO, indicating improved resistance to CO cross-interference. In addition, the Pd–Cu sensor exhibited faster response/recovery behavior, better cycle repeatability, and more stable output after temperature compensation. These results demonstrate that the selected Pd–Cu-sensitive layer can improve the anti-interference capability and sensing stability of the fiber-optic MEMS hydrogen sensor relative to pure Pd under the tested conditions.

Although the selected Pd–Cu-sensitive layer showed improved CO-interference resistance, the Pd:Cu = 7:3 ratio should be regarded as a representative composition for preliminary evaluation rather than an optimized alloy formulation. Further work should include systematic optimization of Cu content, comparison with other Pd-based alloy systems, and detailed thin-film characterization, including quantitative composition verification, surface morphology analysis, phase-structure characterization, and batch-to-batch reproducibility evaluation. In addition, validation under more realistic multi-gas atmospheres, broader temperature ranges, and long-term operating conditions will be necessary to further clarify the relationship between alloy composition, film microstructure, CO-interference resistance, and hydrogen-sensing stability in practical early-warning scenarios.

## Figures and Tables

**Table 1:** Main experimental conditions for gas-sensing measurements.

Parameter	Condition
Chamber volume	Approximately 5 L
Test gases	H_2_/N_2_, CO/N_2_, and N_2_
H_2_ concentration range	0–500 ppm
CO interference concentration	500 ppm
Exposure/purge duration	Approximately 20 min per stage
Test temperature and humidity	25 ± 1 °C, dry-gas condition
Optical spectrum analyzer	YOKOGAWA AQ6370E, 0.02 nm resolution
Wavelength extraction	Minimum wavelength of the selected interference dip
Gas-flow control	Constant inlet flow maintained using gas flowmeters

**Table 2:** Dynamic response and repeatability of the pure Pd and Pd–Cu sensors over three exposure–purge cycles.

Sensor	Response Time t_90_ (min)	Recovery Time t_90_ (min)	Cycle-to-Cycle RSD (%)
Pure Pd	7.6 ± 0.4	5.5 ± 0.3	3.8
Pd–Cu	3.6 ± 0.3	3.1 ± 0.2	2.1

**Table 3:** Temperature calibration parameters and residual-error estimation.

Sensor	Temperature Sensitivity (nm °C^−1^)	R^2^	Maximum Residual Wavelength Error (nm)	Equivalent H_2_ Error (ppm)
pure Pd	0.225	0.9990	<0.05	<0.6
Pd–Cu	0.237	0.9992	<0.05	<0.6

## Data Availability

The data presented in this study are available on request from the corresponding author.

## References

[1] Yang F., Wang T., Deng X., Dang J., Huang Z., Hu S., Li Y., Ouyang M. (2021). Review on hydrogen safety issues: Incident statistics, hydrogen diffusion, and detonation process. Int. J. Hydrogen Energy.

[2] Hu D., Huang S., Wen Z., Gu X., Lu J. (2024). A review on thermal runaway warning technology for lithium-ion batteries. Renew. Sustain. Energy Rev..

[3] Wang Z., Zhu L., Liu J., Wang J., Yan W. (2022). Gas sensing technology for the detection and early warning of battery thermal runaway: A review. Energy Fuels.

[4] Tao Z., Zhou H., Cao Y., Huang X., Wang W., Zhang H. (2026). A review of thermal runaway gases and detection methods for lithium-ion batteries. Therm. Sci. Eng. Prog..

[5] Liu L., Guo C., Wang Y., Guan K., Qin S., Gou X., Sun F., Zhang C., Zhou W., Cai Z. (2026). A comprehensive review of hydrogen sensor for thermal runaway monitoring: Fundamentals, recent advancements, and challenges. Microsyst. Nanoeng..

[6] Xu L., Wang S., Li Y., Li Y., Sun J., Zhao F., Wang H., Wang Y., Xu C., Feng X. (2024). Thermal runaway propagation behavior and gas production characteristics of NCM622 battery modules at different state of charge. Process Saf. Environ. Prot..

[7] Song Y., Jiang X., Lyu N., Lu H., Zhang D., Li H., Jin Y. (2025). Early warning of lithium-ion battery thermal runaway based on gas sensors. eTransportation.

[8] Abdalwareth A., Flachenecker G., Angelmahr M., Schade W. (2023). Optical fiber evanescent hydrogen sensor based on palladium nanoparticles coated Bragg gratings. Sens. Actuators A Phys..

[9] Dai J., Zhu L., Wang G., Xiang F., Qin Y., Wang M., Yang M. (2017). Optical fiber grating hydrogen sensors: A review. Sensors.

[10] Yang M., Dai J. (2014). Fiber optic hydrogen sensors: A review. Photonic Sens..

[11] Ma J., Zhou Y., Bai X., Chen K., Guan B.-O. (2019). High-sensitivity and fast-response fiber-tip Fabry–Pérot hydrogen sensor with suspended palladium-decorated graphene. Nanoscale.

[12] Zhong J., Lu S., Liu S., Chen P., Luo J., Chen Y., Hong G., Xu X., Qu J., Liu L. (2023). Fiber-tip Fabry–Pérot interferometer with a graphene-Au-Pd cantilever for trace hydrogen sensing. Lab Chip.

[13] Xu F., Ma J., Li C., Ma C., Li J., Guan B.-O., Chen K. (2023). Fabry–Pérot cavities with suspended palladium membranes on optical fibers for highly sensitive hydrogen sensing. Molecules.

[14] Xiong C., Zhou J., Liao C., Zhu M., Wang Y., Liu S., Li C., Zhang Y., Zhao Y., Gan Z. (2020). Fiber-tip polymer microcantilever for fast and highly sensitive hydrogen measurement. ACS Appl. Mater. Interfaces.

[15] Coelho L., de Almeida J.M.M.M., Santos J.L., Viegas D. (2015). Fiber optic hydrogen sensor based on an etched Bragg grating coated with palladium. Appl. Opt..

[16] Luo J., Liu S., Chen P., Lu S., Zhang Q., Chen Y., Du B., Tang J., He J., Liao C. (2021). Fiber optic hydrogen sensor based on a Fabry–Pérot interferometer with a fiber Bragg grating and a nanofilm. Lab Chip.

[17] Lee E., Lee J.M., Koo J.H., Lee W., Lee T. (2010). Hysteresis behavior of electrical resistance in Pd thin films during the process of absorption and desorption of hydrogen gas. Int. J. Hydrogen Energy.

[18] Amandusson H., Ekedahl L.-G., Dannetun H. (2000). The effect of CO and O_2_ on hydrogen permeation through a palladium membrane. Appl. Surf. Sci..

[19] Darmadi I., Nugroho F.A.A., Langhammer C. (2020). High-performance nanostructured palladium-based hydrogen sensors—Current limitations and strategies for their mitigation. ACS Sens..

[20] Wang G., Dai J., Yang M. (2021). Fiber-optic hydrogen sensors: A review. IEEE Sens. J..

[21] Jia C., Zhao L., Huang G., Liu L., Wang W., Yang Y., Miao Y. (2023). A review of hydrogen sensors for ECLSS: Fundamentals, recent advances, and challenges. Appl. Sci..

[22] Jewell L.L., Davis B.H. (2006). Review of absorption and adsorption in the hydrogen–palladium system. Appl. Catal. A Gen..

[23] Ollagnier A., Fabre A., Thundat T., Finot E. (2013). Activation process of reversible Pd thin film hydrogen sensors. Sens. Actuators B Chem..

[24] Zhang X., Li X., Zhang X.-P., Peng W. (2025). Fiber optics-mechanics coupling sensor for high-performance hydrogen detection. Photonic Sens..

[25] Huang Y.-W., Tao J., Huang X.-G. (2016). Research progress on F-P interference-based fiber-optic sensors. Sensors.

[26] Ma Z., Song Z., Huang X., Guo T., Yuan W., Chen H., Zhang T., Wang W. (2019). A zero-cross detection algorithm for cavity-length interrogation of fiber-optic Fabry–Perot sensors. Sensors.

